# *In Vitro* Antiophidian Mechanisms of *Hypericum brasiliense* Choisy Standardized Extract: Quercetin-Dependent Neuroprotection

**DOI:** 10.1155/2013/943520

**Published:** 2013-12-31

**Authors:** Cháriston André Dal Belo, Ana Paula de Bairros Lucho, Lúcia Vinadé, Leandro Rocha, Hildegardo Seibert França, Sérgio Marangoni, Léa Rodrigues-Simioni

**Affiliations:** ^1^CIPBIOTEC, Federal University of Pampa, (UNIPAMPA), Campus São Gabriel, 97300-000 São Gabriel, RS, Brazil; ^2^Laboratory of Natural Products Technology, Federal University Fluminense, Faculty of Pharmacy, 24241-002 Niterói, RJ, Brazil; ^3^Federal Institute of Espírito Santo, Campus Vila Velha, 29106-010 Vila Velha, Espírito Santo, Brazil; ^4^LAQUIP, Department of Biochemistry, Institute of Biology, State University of Campinas (UNICAMP), P.O. Box 6109, 13083-970 Campinas, SP, Brazil; ^5^Department of Pharmacology, Faculty of Medical Sciences, State University of Campinas (UNICAMP), P.O. Box 6111, 13083-970 Campinas, SP, Brazil

## Abstract

The neuroprotection induced by *Hypericum brasiliense* Choisy extract (HBE) and its main active polyphenol compound quercetin, against *Crotalus durissus terrificus* (Cdt) venom and crotoxin and crotamine, was enquired at both central and peripheral mammal nervous system. Cdt venom (10 **μ**g/mL) or crotoxin (1 **μ**g/mL) incubated at mouse phrenic nerve-diaphragm preparation (PND) induced an irreversible and complete neuromuscular blockade, respectively. Crotamine (1 **μ**g/mL) only induced an increase of muscle strength at PND preparations. At mouse brain slices, Cdt venom (1, 5, and 10 **μ**g/mL) decreased cell viability. HBE (100 **μ**g/mL) inhibited significantly the facilitatory action of crotamine (1 **μ**g/mL) and was partially active against the neuromuscular blockade of crotoxin (1 **μ**g/mL) (data not shown). Quercetin (10 **μ**g/mL) mimicked the neuromuscular protection of HBE (100 **μ**g/mL), by inhibiting almost completely the neurotoxic effect induced by crotoxin (1 **μ**g/mL) and crotamine (1 **μ**g/mL). HBE (100 **μ**g/mL) and quercetin (10 **μ**g/mL) also increased cell viability in mice brain slices. Quercetin (10 **μ**g/mL) was more effective than HBE (100 **μ**g/mL) in counteracting the cell lysis induced by Cdt venom (1 and 10 **μ**g/mL, resp.). These results and a further phytochemical and toxicological investigations could open new perspectives towards therapeutic use of *Hypericum brasiliense *standardized extract and quercetin, especially to counteract the neurotoxic effect induced by snake neurotoxic venoms.

## 1. Introduction

An estimated 5.4-5.5 million people are bitten by snakes each year, resulting in about 400.000 amputations and about 125.000 deaths [1, 2]. The problem of human suffering by snake bite is actually so relevant that WHO has included it in the list of neglected tropical diseases in April, 2009 [3].

Snake venoms embody a complex mixture of toxic enzymes and proteins, such as myotoxins, neurotoxins, cytotoxins, hemorrhagic metalloproteases, clotting serineproteases, and others [4]. Among all snake venoms, the crotalic is one of the most neurotoxic, in which systemic effects reside primarily in the peripheral neurotoxicity. However, when injected directly on CNS of mammals it can induce convulsion and death [5]. Among other symptoms, the neurotoxicity induced by *Crotalus* poisoning in both central and peripheral nervous system is mainly related to the presence in the venom of the toxins crotoxin [6] and crotamine [7]. Thus, the search of novel venom inhibitors is therefore relevant, being natural or synthetic, in order to complement the current serum therapy and to neutralize the remaining damages of snake envenomation.

*Hypericum brasiliense* is an annual cycle plant, recurrent in the southern and southeastern Brazil, known by the common names of “milfurada”, “milfacadas,” and “alecrim bravo” [8, 9]. *H. brasiliense* extract has shown anti-inflammatory and analgesic [10] activities, with contradictory signs on the CNS [11] and protection of mice against lethality of *Bothrops jararaca* venom [12].

The present work demonstrates the ability of *Hypericum brasiliense* standardized extract and quercetin to counteract neurodegenerative insults induced by Cdt venom in brain and muscles preparations. In addition, it is shown that the major neurotoxic components of the *Crotalus durissus terrificus* venom, crotoxin and crotamine, also had their effects prevented in the neuromuscular paralysis at mouse nerve-muscle preparations.

## 2. Experimental

### 2.1. Reagents and Venom

All chemicals and reagents used were of the highest purity and were obtained from Sigma, Aldrich, Merck or BioRad. *Crotalus durissus terrificus* venom, crotamine and crotoxin were donated by Dr. S. Marangoni (UNICAMP) and quercetin by Dr. L. Rocha (UFF).

### 2.2. Animals

Adult Swiss white mice (28–35 g) from both sexes were supplied by the Multidisciplinary Center for Biological Investigation (CEMIB) at UNICAMP and by the animal facility from Universidade Federal de Santa Maria (UFSM). The animals were housed at 25°C with access *ad libitum* to food and water. These studies have been done in accordance with the guidelines of the Brazilian College for Animal Experimentation (COBEA).

### 2.3. Plant Material

*Hypericum brasiliense* leaves were collected in the city of Nova Friburgo, RJ, Brazil, in 2001. A voucher specimen (n°19980) has been deposited at the herbarium of the Museu Nacional, Universidade Federal do Rio de Janeiro, Brazil.

### 2.4. Chemical Analysis

The preparation of *H. brasiliense* EtOH extract (HBE) and detection of its chemical composition were carried out as described elsewhere [13]. Briefly, the chemical analysis was performed with a Liquid Chromatograph (GBC Scientific Equipment LLC, Hampshire, IL, USA), equipped with a Nucleosil MN 120-5 C_18_ silica column (Macherey-Nagel Inc., Bethelehem, PA, USA). The elution was made at room temperature using a linear gradient from 10–60% of acetonitrile in trifluoroacetic acid (0.05% v/v) at a flow rate of 1.0 mL/min in 30 minutes. Peaks were monitored at 254 nm in order to quantify the flavonoid quercetin.

### 2.5. Hippocampal Slices Preparation

Mice were decapitated, the brains removed immediately, and the hippocampus dissected on ice and humidified in cold HEPES-saline buffer gassed with O_2_ (124 mm NaCl, 4 mM KCl, 1.2 mM MgSO_4_, 12 mM glucose, 1 mM CaCl_2_, and 25 mM HEPES pH 7.4). Hippocampal slices were obtained according to Vinadé & Rodnight [14], briefly: a Mcilwain tissue chopper was used to obtain the slices (0.4 mm) that were separated and preincubated at 37°C for 30 min in microwell plates filled with HEPES saline (200 *μ*L/slice). Subsequently, fresh medium was replaced (200 *μ*L/slice) for control condition and treatments with Cdt (1, 5 and 10 *μ*g/mL), HBE (100 *μ*g/mL), HBE + Cdt, quercetin (10 *μ*g/mL), and quercetin + Cdt and incubated for 1 hour (37°C).

### 2.6. Hippocampal Slices Viability

Immediately after incubation with treatments, slices were assayed for a 3-(4,5-dimethylthiazol-2-yl)-2,5-diphenyltetrazolium bromide (MTT) test (0.05% in HEPES-saline) for 30 min (37°C) [15]. The MTT is converted into a purple formazan product after cleavage of the tetrazolium ring by mitochondrial dehydrogenases. Formazan was dissolved by the addition of DMSO, resulting in a colored compound whose optical density (*λ* = 550 nm) was measured in an ELISA reader equipment [16].

### 2.7. Phrenic Nerve-Diaphragm Preparation

Whole diaphragms along with the phrenic nerves were removed from mice killed by carbon dioxide (CO_2_) and exsanguinated. Both hemidiaphragms were mounted essentially as described for dal Belo et al. [17]. The preparations were suspended under a constant tension of 5 g in a 5 mL organ bath containing aerated (95%O_2_–5%CO_2_) Tyrode solution (pH 7.4, 37°C) of the following composition (mM): NaCl 137, KCl 2.70, CaCl_2_ 1.80, MgCl_2_ 0.490, NaH_2_PO_4_ 0.420, NaHCO_3_ 11.9, and glucose 11.1. Supramaximal stimuli (0.1 Hz, 0.2 ms) delivered by a Grass S4 electronic stimulator (Grass Instrument Co., Quincy, MA, USA) were applied through electrodes placed around the motor nerve, corresponding to an indirect stimulation.

### 2.8. Statistical Analysis

The results were expressed as the mean ± SEM and were compared statistically using ANOVA for repeated measures. A  *P* value < 0.05 indicated significance.

## 3. Results

**Figure 1:**
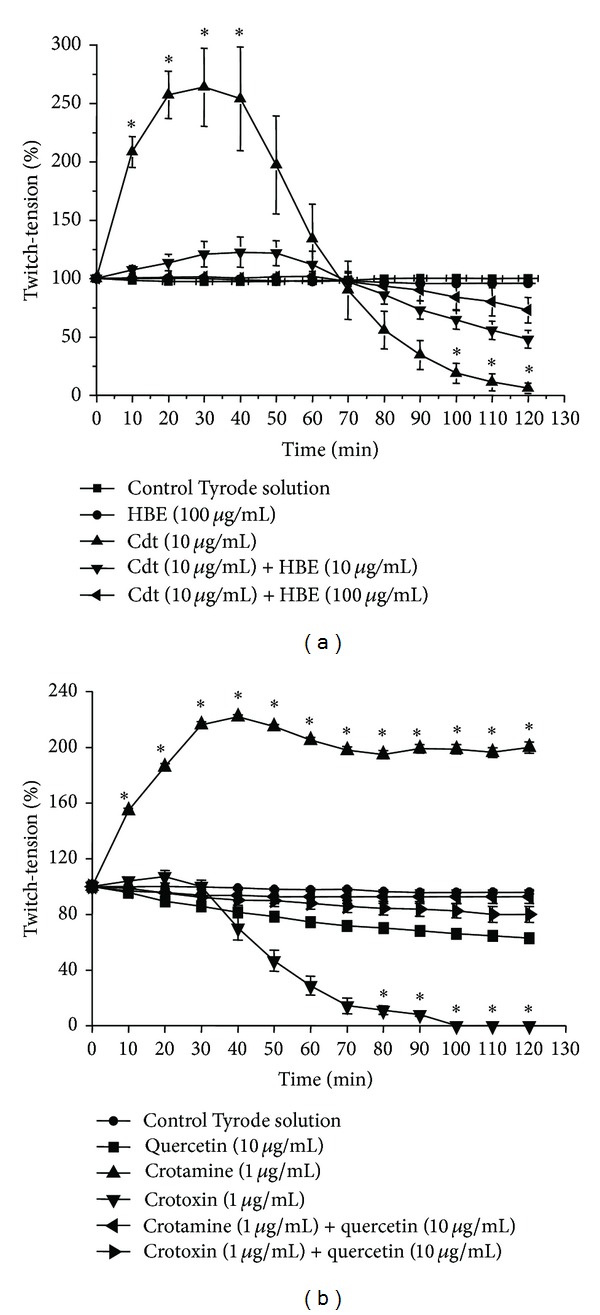
Neutralizing activity of *H. brasiliense* ethanolic plant extract (HBE) against Crotalic venom, crotoxin and crotamine at mouse phrenic nerve-diaphragm preparations. Panel (a) shows the inhibitory effect of HBE (10 and 100 *μ*g/mL) against *C. d. terrificus* venom (10 *μ*g/mL), crotoxin and crotamine. In Panel (b) effect of Quercetin (10 and 100 *μ*g/mL) against Cdt (10 *μ*g/mL), crotoxin (1 *μ*g/mL), and crotamine (1 *μ*g/mL). When HBE was applied alone in the organ-bath no alteration in the twitch-tension was observed. On both graphs control Tyrode solution lines show no alteration of normal nerve-muscle activity. The points on the graphs represent the mean ± S.E.M. of five experiments. On (b) note that quercetin mimicked the protective effect induced by HBE. HBE: *Hypericum brasiliense* standardized extract *P < 0.05 compared to control Tyrode.

HBE was shown to be rich in flavonoids derivatives such as kaempferol, quercetin, and quercetin glycosides (quercitrin, isoquercitrin, guaijaverin, and hyperoside) [13]. The selective extraction of polyphenol compounds in HBE resulted, after hydrolysis, in not less than 6,7% of total flavonoids, expressed as quercetin. Incubation of mouse phrenic nerve-diaphragm preparation (PND) with Tyrode solution did not induce alterations in basal muscle twitch tension during 120 min recordings (*n* = 5, Figure 1). When *Crotalus durissus terrificus* venom (Cdt, 10 *μ*g/mL) was added to (PND) preparation there was an increase of 160% in the muscle twitch tension followed by an irreversible and complete neuromuscular blockade after 70 min (*n* = 5, Figure 1). Incubation of PND preparation with HBE (10 and 100 *μ*g/mL) produced no alteration in the amplitude of muscle twitch tension (*n* = 5), during 120 min observation. However, when preparations were assayed with a mixture of HBE (50 *μ*g/mL and 100 *μ*g/mL) and Cdt venom (10 *μ*g/mL) previously incubated during 30 min at 37°C, the characteristic neuromuscular blockade was prevented in 75% with the highest concentration of the extract (Figure 1(a), *n* = 5, *P* < 0.05). The assay of the myotoxin crotamine (1 *μ*g/mL) alone at PND preparations induced a significative increase of muscle twitch tension (~150%), that was maximum at 30 min (*P* < 0.05, *n* = 6, Figure 1(b)). On the contrary, the addition of the PLA_2_ neurotoxin crotoxin isolated (1 *μ*g/mL) at PND preparations caused a progressive and irreversible neuromuscular blockade during 120 min recordings (*P* < 0.05, *n* = 6). The assay of HBE (100 *μ*g/mL) + crotamine (1 *μ*g/mL) or crotoxin (1 *μ*g/mL), previously incubated for 30 min at 37°C, inhibited 100% of the facilitatory actions induced by crotamine and 85% of the neuromuscular blockade caused by crotoxin (1 *μ*g/mL), respectively, in 120 min recordings (*n* = 5, *P* < 0.05, data not shown). When quercetin (10 *μ*g/mL) was incubated alone, there was a maximum decrease of muscle twitch tension of 20 ± 0.5% in 120 min recordings, although not significative (Figure 1(b), *P* > 0.05 compared to the control Tyrode). The addition of quercetin (10 *μ*g/mL) with crotamine (1 *μ*g/mL) or crotoxin (1 *μ*g/mL) previously incubated for 30 min at 37°C showed a more potent antineurotoxic activity when compared to the HBE. This increased potency of quercetin compared to HBE must be due to a higher effective concentration of the flavonoid when compared to the whole extract (~7%). Quercetin was able to completely inhibit the facilitatory actions of crotamine (1 *μ*g/mL) and decreased in 80 ± 5% the neuromuscular blockade induced by crotoxin (1 *μ*g/mL) (*n* = 5, *P* < 0.05, Figure 1(b)).

**Figure 2:**
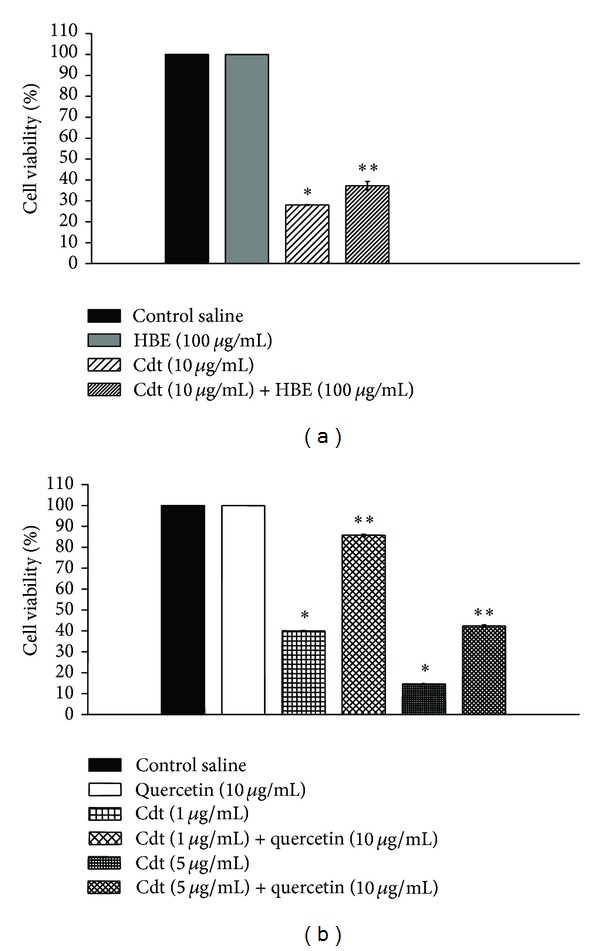
Effects of Cdt on the viability of hippocampal slices. (a) Hippocampal slices were incubated with HBE (100 *μ*g/mL) in the presence or absence of Cdt (10 *μ*g/mL) during 1 hour. (b) Hippocampal slices were incubated during 1 hour with Quercetin (10 *μ*g/mL) in the presence or absence of Cdt (1 and 5 *μ*g/mL). Cell viability was measured by MTT test. Values are expressed as % of control, which was defined as untreated slices (values are means ± S.E.M., *n* = 4). On (b) note that quercetin mimicked the HBE protective activity. HBE: *Hypericum brasiliense* standardized extract**P < 0.05 compared to control*.

The effect of HBE (100 *μ*g/mL) or quercetin (10 *μ*g/mL) alone was accessed at central nervous system (CNS) through hippocampal slices. In this set of experiments the cell viability was not modified after 1 h incubation with both vegetal extract and the pure flavonoid. On the other hand, the incubation of Cdt venom in doses of (1, 5, and 10 *μ*g/mL) significantly decreased the cell viability (40 ± 3, 14 ± 1 and 28 ± 1%, *n* = 3, *P* < 0.05, resp.) (Figures 2(a) and 2(b)). The addition of HBE (100 *μ*g/mL) with Cdt (10 *μ*g/mL) to the slices produced a slight protection compared to the control Cdt (*n* = 3, *P* < 0.05) (Figure 2(a)). However, the blend of quercetin (10 *μ*g/mL) and Cdt (1 *μ*g/mL or 5 *μ*g/mL), significantly inhibited the cell lysis showing a protection in the order of 46 ± 2% and 12 ± 1%, *n* = 4, *P* < 0.05, respectively (Figure 2(b)). The results in hippocampal slices confirm the HBE and quercetin potential role in the neuroprotection against Cdt poisoning. Therefore, the difference in potency between HBE and quercetin must also be related to the less amount of the flavonoid in the extract.

## 4. Discussion

In this work we described for the first time the effectiveness of the *H. brasiliense* extract (HBE) and its marked compound quercetin, against the neuromuscular paralysis induced by *Crotalus durissus terrificus* snake venom (Cdt), crotoxin, and crotamine at mouse phrenic nerve-diaphragm preparations. Also, the effectiveness of HBE and quercetin was validated, to counteract the deleterious effects induced by *C. d. terrificus* venom, on cell viability of mouse brain slices. Crotalus venom induces neurotoxicity, coagulation disorders, systemic myotoxicity, and acute renal failure [18], with possible additional heart and liver damage [19]. This venom is a mixture of enzymes, toxins (crotoxin, crotamine, gyroxin, and convulxin), and several other peptides [19]. The characteristic pathophysiological pictures of neurotoxicity and systemic myotoxicity associated with *C. d. terrificus* envenomation are mainly related to the presence in the venom of crotoxin, a neurotoxic PLA_2_ heterodimeric complex, which causes progressive paralysis, and in high concentrations myonecrosis [20, 21]. At nerve terminals, crotoxin induces triphasic alterations in the mean quantal content of transmitter release with a slow and progressive decrease of presynaptic release of the neurotransmitter acetylcholine that results in complete neuromuscular blockade [22, 23]. At mammal central nervous system, the injection of Cdt venom induces seizures [5], which is mainly associated with the presence of crotoxin [24]. At brain synaptosomes, crotoxin has also shown the ability of inhibiting L-glutamate and gamma aminobutyric acid (GABA) uptake [25]. Crotamine is the second major toxin in the Cdt venom; it is a basic, low molecular weight myotoxin devoid of PLA_2_ activity [26], with a specific action on voltage-sensitive sodium channels of muscles [27] and brain cells [28].

Flavonoids are plant secondary metabolites that embrace a wealth of possibilities of hydrogen bonding arranged around a relatively small carbon skeleton, capable of interacting with molecular targets [29]. In the *H. brasiliense* extract, the flavonoid quercetin and its derivatives were shown to be the major secondary metabolites in the plant. Quercetin and several of its glycosides are the most often encountered flavonoids in anti-snake venom plants where *Albizia lebbeck*, *Achillea millefolium*, *Euphorbia hirta*, *Camellia sinensis*, and *Casearia sylvestris* are some examples. Flavonoids have been reported as snake venom phospholipase A_2_ inhibitors [30].

Recent studies revealed that the treatment of the snake venom PLA_2_ isoform from *Crotalus durissus cascavella* snake venom with the flavonoid quercetin produced a decrease in the pharmacological activity of the neurotoxin by inducing alterations in the secondary but not in tertiary structure composition of the molecule [31]. As discussed above, flavonoids have the ability of binding to biological polymers (e.g., enzyme inhibiting activities). Therefore, snake PLA_2_ catalyzed the production of lysophospholipids and fatty acids that are involved in membrane damage [21]. We suggest that, in the case where biological activity is enzyme-dependent, the HBE antineurotoxic activity would involve the inactivation of PLA_2_ activity by quercetin. However, the possibility that the HBE acts through a mechanistic intervention rather than an *in vitro* direct physical interaction with the venom is also a reasonable idea. This is likely to be the mode of action of many polyphenolic compounds found in plant extracts, which probably explains many of the “protective” effects of plant extracts when they are preincubated with venom before administration to the biological assay [32, 33].

Flavonoids derived from plants or tea extracts also affect acetylcholine release, muscle contraction, or neuromuscular junction activity [34]. In this regard, the muscle-type nicotinic acetylcholine receptor consists of *α*1*β*1*ε*, in adult tissue [35]. It was found that quercetin inhibits the muscle type nicotinic acetylcholine receptor, by binding on the *γ* or *ε* subunities, which is a characteristic of a noncompetitive inhibitor [36]. Crotoxin also stabilizes the postsynaptic membrane of *Torpedo marmorata* by binding in non-ACh biding sites [37]. Hence, these similarities in terms of binding sites would strengthen the hypothesis of a site-direct antagonism between quercetin and crotoxin at nerve terminals. In addition, quercetin actively participates in intracellular signaling, inhibiting phosphatidylinositol-3 kinase, protein kinase C, xanthine oxidase, and NADPH diaphorase [34]. In massive cellular insults like ischemia, involving metabolic failure, loss of Ca^2+^ homeostasis, and excitotoxicity, scavenger activity or one-target antioxidant mechanisms (NMDA receptor blockers, chain-breaking vitamin E, or pure scavenger molecules such as boldine) may fail to protect cells from free radical damage. Current explanation for the neuroprotective effect of quercetin is its antioxidant capacity and its ability to scavenge free radicals [34]. At moment there is no evidence that snake venoms induce cellular insults to increase free radicals in nerve terminals. However, the actions of Cdt venom on cell viability of brain slices is likely to be devoid to the presence of crotoxin and crotamine that ultimately account for the increase of excitatory neurotransmitters [22], resulting in excitotoxicity [38]. The decrease in neurotransmitter uptake by crotoxin is calcium independent [25], and quercetin potentiates neuronal excitability by increasing neuronal firing rates [39]. Ultimately, excitotoxicity is a result of synaptic dysfunction processes, which involves the excessive glutamate receptor activation and neuronal degeneration [38]. Based on the above considerations we suggest that the mechanism of the benefit of quercetin on snake venom-induced neuronal cellular death is complex and beyond the inhibition of presynaptic activity of snake PLA_2_, and structural modifications, which may affect neurotransmitter uptake, involve the maintenance of neuronal mitochondrial transmembrane electric potential which would decrease the overstimulation of glutamate receptors [34]. However, in the case of crotamine, a direct inhibition of voltage-gated sodium channels by quercetin seems to be a coherent explanation [40].

Further investigation on *Hypericum brasiliense* isolated compounds will strengthen the understanding of its antiophidian activity. Preclinical assays, including safety assessment protocols, could also open the way towards therapeutic use of *Hypericum brasiliense* especially when neurotoxic venoms are involved.
